# Impact of an antibiotic stewardship initiative on urgent-care respiratory prescribing across patient race, ethnicity, and language

**DOI:** 10.1017/ice.2023.258

**Published:** 2023-12-11

**Authors:** Allan M. Seibert, Adam L. Hersh, Payal K. Patel, Lauri A. Hicks, Nora Fino, Valoree Stanfield, Edward A. Stenehjem

**Affiliations:** 1 Division of Infectious Diseases, Intermountain Health, Salt Lake City, Utah; 2 Division of Infectious Diseases, Department of Pediatrics, University of Utah School of Medicine, Salt Lake City, Utah; 3 Division of Healthcare Quality Promotion, Centers for Disease Control and Prevention, Atlanta, Georgia; 4 Division of Epidemiology, Department of Internal Medicine, University of Utah, Salt Lake City, Utah; 5 Office of Patient Experience, Intermountain Health, Salt Lake City, Utah; 6 Division of Infectious Diseases, University of Colorado School of Medicine, Aurora, Colorado

## Abstract

We conducted a post hoc analysis of an antibiotic stewardship intervention implemented across our health system’s urgent-care network to determine whether there was a differential impact among patient groups. Respiratory urgent-care antibiotic prescribing decreased for all racial, ethnic, and preferred language groups, but disparities in antibiotic prescribing persisted.

Most antibiotic prescriptions in the United States occur in the outpatient setting, and up to 30% may be unnecessary.^1^ Urgent care remains one of the fastest growing sites of outpatient care delivery in the United States, with encounter volumes increasing by 50% or more in recent years, and this trend is projected to continue.^2^ Urgent-care encounters result in more antibiotic prescriptions overall and more unnecessary antibiotic prescriptions compared with other outpatient settings.^3^ To improve antibiotic prescribing, we implemented an antibiotic stewardship intervention across the Intermountain Health urgent-care network from July 1, 2019, through June 30, 2020.^4^ The intervention was based on the Centers for Disease Control and Prevention (CDC) Core Elements of Outpatient Antibiotic Stewardship^5^ and included English and Spanishlanguage education focused on urgent-care clinicians and patients, electronic health record (EHR) tools to assist clinicians in ordering antibiotic prescriptions correctly and more efficiently documenting encounters, a transparent clinician benchmarking dashboard, and media targeting patients and clinicians. Occurring in parallel and independent of the development of the stewardship intervention, Intermountain Health urgent-care leadership introduced an antibiotic prescribing quality measure^6^ with a target of <50% respiratory encounters receiving an antibiotic.

Antibiotic prescribing rates have previously been shown to differ by patient race, ethnicity, and preferred language; understanding these possible healthcare inequities is a health system priority.^7,8^ Addressing health equity was not an explicit consideration when designing our intervention. Subsequent organizational efforts to identify disparities in care delivery across our system, possibly representing inequitable care, revealed prescribing differences across patient race, ethnicity, and preferred language prior to the intervention.^9^ To further understand these differences, we conducted a post hoc analysis of our intervention to assess whether its effect differed by patient race, ethnicity, or preferred language and to determine if disparities in antibiotic prescribing between groups persisted.

## Methods

Intermountain Health is a nonprofit, integrated, healthcare delivery system that operated 38 urgent-care clinics during the study period from July 1, 2018, to June 30, 2020. Pediatric and adult patients were included in our study. We used a before-and-after, quality-improvement study design to evaluate the effect of the systemwide antibiotic stewardship intervention on average monthly antibiotic prescribing rates for urgent-care respiratory encounters. We compared a 12-month baseline period (July 2018–June 2019) to a 12-month intervention period (July 2019–June 2020) using an interrupted time-series (ITS) model^10^ to assess the effect of the intervention on antibiotic prescribing by patient race, ethnicity, and preferred language. Population characteristics categories included race (White vs Peoples other than White), ethnicity (Hispanic vs non-Hispanic), and preferred speaking language (English vs Spanish). American Indian or Alaskan Native, Asian, Black or African American, Multiple, Native Hawaiian or Pacific Islander, Other, and patients declining or failing to provide a preferred race were included in the Peoples other than White category for the purposes of our analysis due to the demographics of our health system. Separate models were performed for each characteristic and for comparison between groups within each characteristic category. Characteristics were self-reported by patients at the time of the encounter and were extracted from the EHR.

## Results

Patient characteristics for respiratory urgent-care encounters during the baseline and intervention periods were similar: mean age, 30.0 versus 30.7 years; White race 92.0% versus 91.2%; non-Hispanic 87.4% versus 86.0%; English language preferred 98.0% versus 97.7%. Comprehensive demographic information for the baseline and intervention periods is available in Table 1. Overall, 207,047 respiratory urgent-care encounters (41.9%) occurred during the baseline period and 183,726 (39.0%) took place during the intervention period. Furthermore, 98,867 respiratory urgent-care encounters (47.8%) were associated with an antibiotic prescription during the baseline period and 61,243 (33.3%) during the intervention period.

**Figure 1.**
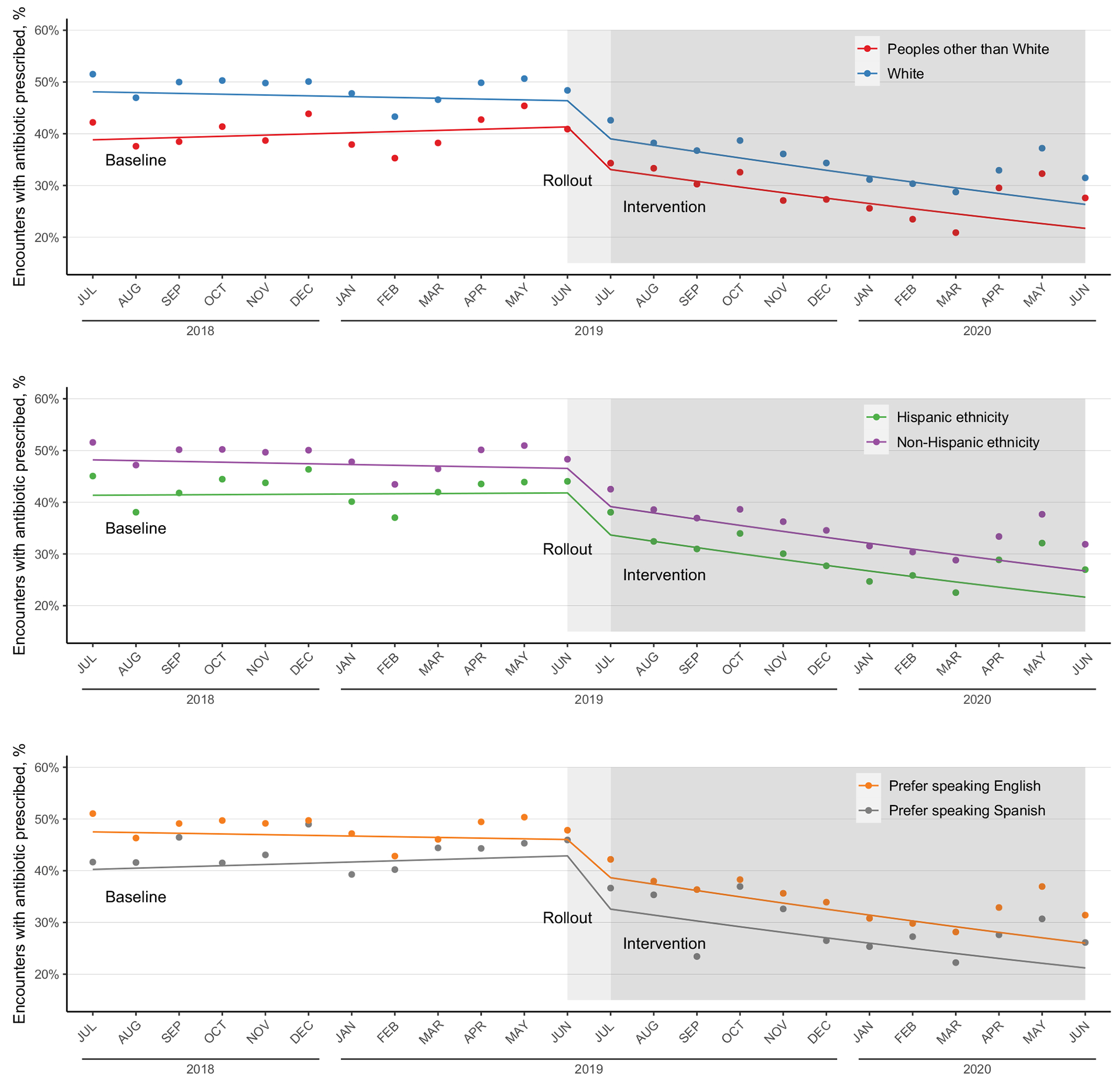
Fitted interrupted time-series models for urgent-care respiratory-encounter antibiotic prescribing during the baseline, rollout, and intervention periods, by race, ethnicity, and preferred language. Individual points represent the observed fraction of encounters receiving an antibiotic in a particular month, and the solid-color lines represent the fitted interrupted time series model among groups. All groups exhibited similar seasonal variability during the study.

All evaluated race, ethnicity, and preferred language categories demonstrated a decrease in respiratory antibiotic prescribing during the intervention compared to the baseline. However, differences in prescribing between groups persisted over the course of our study. Regarding race, between the baseline and intervention periods, antibiotic prescribing decreased among White patients from 92,206 encounters (48.4%) to 56,825 encounters (33.9%) and among Peoples other than White patients from 5,460 encounters (39.9%) to 3,583 encounters (27.0%). Regarding ethnicity, antibiotic prescribing decreased among non-Hispanic patients from 87,909 encounters (48.4%) to 54,210 (34.1%) encounters and among Hispanic patients from 9,243 encounters (42.1%) to 5,959 encounters (28.0%). Regarding preferred language, antibiotic prescribing decreased among patients who preferred English from 97,230 encounters (47.9%) to 60,123 encounters (33.5%) and among patients who preferred Spanish from 1,259 encounters (43.2%) to 844 encounters (27.7%) (Fig. 1). Using ITS analysis, the impact of the intervention was not different between studied groups: White people (odds ratio [OR], 0.95; 95% confidence interval [CI], 0.94–0.96) versus Peoples other than White (OR, 0.95; 95% CI, 0.94–0.96; *P* = .13); non-Hispanic ethnicity (OR, 0.95; 95% CI, 0.94–0.96) versus Hispanic (OR, 0.95; 95% CI, 0.93–0.97; *P* =.25); and those who preferred speaking English (OR, 0.95; 95% CI, 0.94–0.96) versus those who preferred Spanish (OR, 0.95; 95% CI, 0.90–0.99; *P* = .56).

## Discussion

We noted a significant decrease in urgent-care respiratory-encounter antibiotic prescriptions for all patients regardless of race, ethnicity, or preferred language. We did not observe a differential impact of the intervention across these categories, but disparities in antibiotic prescribing between groups persisted. These results suggest that our stewardship intervention decreased respiratory urgent-care antibiotic prescribing for all groups but may not have influenced underlying factors contributing to the differences in antibiotic prescribing between groups. A significant limitation of our study was the small numbers of Peoples other than White race, Hispanic patients, and patients who preferred speaking Spanish. This limited the categories of our analysis and could have resulted in a lack of power to identify a differential impact of the intervention. Patients aged ≥18 years comprised nearly 70% of our study population; however, we did not evaluate the impact of our intervention among racial, ethnic, and preferred language categories separately for adult and pediatric patients. Minoritized pediatric and adult patients may have experienced the intervention differently. Our study population was predominantly White, non-Hispanic, and preferred speaking English, consistent with the demographics of our service area. This factor likely limits the generalizability of our findings.

Critically, we were unaware of the differences in antibiotic prescribing between groups prior to the development of our intervention and did not consider these differences in its design. These differences would have been an initial factor to consider had we tailored our intervention to also attenuate disparities between groups. Structured interviews with providers and patients may have revealed whether delayed antibiotic prescriptions or symptomatic therapies were viewed differently by some groups. Education materials could have then been customized to address concerns noted in different groups. Patient race, ethnicity, or preferred speaking language categories were not included in our antibiotic prescribing dashboard during the intervention, but they have since been integrated. Examining outcomes of interest by these and other patient characteristics that may be associated with health equity is now standard across our organization. Although our analysis suggests that disparities may have remained similar between studied groups and did not worsen, further study is required to understand why these disparities appear to have persisted. Examining clinic and provider variability across different patient characteristics along with clinic rurality, patient education level, or other forms of structural vulnerability indices may reveal further opportunities to iterate upon our intervention and diminish disparities between patient groups while continuing to decrease inappropriate antibiotic prescribing. Our experience highlights the importance of not only examining interventions via factors related to health equity such as race, ethnicity, and language but also the need to design interventions that address the needs of all patient populations, especially those historically marginalized in healthcare delivery.

## Figures and Tables

**Table 1.** Patient Characteristics for Intermountain Health Respiratory Urgent-Care Encounters during the Baseline and Intervention Periods

Characteristic	Baseline (July 1, 2018–June 30, 2019), No. (%)^a^	Intervention (July 1, 2019–June 30, 2020), No. (%)^a^
**Age**		
Patient age, mean y (SD)	30.0 (21.4)	30.7 (20.8)
Patient age <18 y	67,504 (32.6)	53,403 (29.1)
**Race**		
American Indian or Alaska Native	1,376 (0.7)	1,248 (0.7)
Asian	3,239 (1.6)	2,989 (1.6)
Black or African American	2,456 (1.2)	2,302 (1.3)
Multiple	513 (0.3)	468 (0.3)
Native Hawaiian or Pacific Islander	2,630 (1.3)	2,496 (1.4)
Other, declined, or not provided	6,249 (3.0)	6,660 (3.6)
White race	190,584 (92.0)	167,563 (91.2)
**Sex**		
Female	117,602 (56.8)	103,626 (56.4)
Male	89,445 (43.2)	80,100 (43.6)
**Ethnicity**		
Hispanic ethnicity	21,931 (10.8)	21,316 (11.8)
Not Hispanic	180,925 (87.4)	158,013 (86.0)
Other, declined, or not provided	4,191 (2.0)	4,397 (2.4)
**Language**		
English language preferred	202,985 (98.0)	179,471 (97.7)

Note. SD, standard deviation.

a Data are no. (%) unless otherwise specified.
